# Molecular Epidemiology and Transmission Dynamics of Recent and Long-Term HIV-1 Infections in Rural Western Kenya

**DOI:** 10.1371/journal.pone.0147436

**Published:** 2016-02-12

**Authors:** Clement Zeh, Seth C. Inzaule, Pascale Ondoa, Lillian G. Nafisa, Alex Kasembeli, Fredrick Otieno, Hilde Vandenhoudt, Pauli N. Amornkul, Lisa A. Mills, John N. Nkengasong

**Affiliations:** 1 US Centers for Disease Control and Prevention, Division of HIV/AIDS Prevention (CDC), Kisumu, Kenya; 2 Kenya Medical Research Institute (KEMRI)/CDC Research and Public Health Collaboration, Kisumu Field Research Station, Kisumu, Kenya; 3 Amsterdam Institute of Global Health and Development (AIGHD), Department of Global Health of the Academic Medical Center, Amsterdam, The Netherlands; 4 Institute of Tropical Medicine (ITM), Antwerp, Belgium; 5 Division of Global HIV and Tuberculosis, Center for Global Health, CDC Atlanta, Georgia, United States of America; University of Athens, Medical School, GREECE

## Abstract

**Objective:**

To identify unique characteristics of recent versus established HIV infections and describe sexual transmission networks, we characterized circulating HIV-1 strains from two randomly selected populations of ART-naïve participants in rural western Kenya.

**Methods:**

Recent HIV infections were identified by the HIV-1 subtype B, E and D, immunoglobulin G capture immunoassay (IgG BED-CEIA) and BioRad avidity assays. Genotypic and phylogenetic analyses were performed on the *pol* gene to identify transmitted drug resistance (TDR) mutations, characterize HIV subtypes and potential transmission clusters. Factors associated with recent infection and clustering were assessed by logistic regression.

**Results:**

Of the 320 specimens, 40 (12.5%) were concordantly identified by the two assays as recent infections. Factors independently associated with being recently infected were age ≤19 years (*P* = 0.001) and history of sexually transmitted infections (STIs) in the past six months (*P* = 0.004). HIV subtype distribution differed in recently versus chronically infected participants, with subtype A observed among 53% recent vs. 68% chronic infections (p = 0.04) and subtype D among 26% recent vs. 12% chronic infections (p = 0.012). Overall, the prevalence of primary drug resistance was 1.16%. Of the 258 sequences, 11.2% were in monophyletic clusters of between 2–4 individuals. In multivariate analysis factors associated with clustering included having recent HIV infection *P* = 0.043 and being from Gem region *P* = 0.002.

**Conclusions:**

Recent HIV-1 infection was more frequent among 13–19 year olds compared with older age groups, underscoring the ongoing risk and susceptibility of younger persons for acquiring HIV infection. Our findings also provide evidence of sexual networks. The association of recent infections with clustering suggests that early infections may be contributing significant proportions of onward transmission highlighting the need for early diagnosis and treatment as prevention for ongoing prevention. Larger studies are needed to better understand the structure of these networks and subsequently implement and evaluate targeted interventions.

## Introduction

Identification of recent or acute infections and their role in driving the HIV epidemic is crucial in understanding the epidemic dynamics and in guiding the deployment of appropriate control strategies[1–3]. While long-term infections form important reservoirs for subsequent infections, it has been shown that the risk of infectivity is higher in the recently infected cases due to high viral load during the early stages of HIV infection[1]. Moreover recently infected persons are also less likely to be aware of their HIV diagnosis. Previous studies in Europe have suggested that 25–50% of transmissions among men who have sex with men (MSM) and up to 2% among heterosexuals occur during primary infection[4,5]. There is however a paucity of data from injection drug users despite the high risk of infection among this group. The high risk of transmission in primary infection highlights the importance of epidemiologic investigations for recent infection to help identify and provide early intervention strategies to those most-at-risk persons. While such research has been shown to provide vital public health information, few data have been obtained from Africa despite the high prevalence and incidence rates within the region.[6,7]

Further elucidation of the epidemic dynamics requires the identification and characterization of transmission networks representing important reservoirs of infection.[8] Molecular tools to study linkages between viruses can be used to provide an in-depth understanding of transmission dynamics among different sexual contact groups.[9] The characteristics associated with a sexual transmission network in a given location are essential in determining both the short and long-term equilibrium of the disease prevalence and providing information for effective target-specific intervention strategies. Previous studies in sub-Saharan Africa have led to a common belief that the epidemic in this region is mainly of heterosexual nature with a structure that involves the sex workers or other high-risk groups with subsequent diffusion to the general population through marriage or other stable types of partnership[8]. However, few studies have documented this pattern and while common policies have targeted key players in such a structure, the rate of the epidemic has continued to soar in some regions. Hence a re-assessment of this model is necessary to identify areas of linkage that might provide clues to effective intervention strategies.

In the current study, we determine the levels and characteristics of recent infection as well as the dynamics of transmission networks from two surveys in two populations in rural western Kenya. We further investigate the viral diversity in this region, taking into account the differences between the long-term and recent infections (as determined by the laboratory tests) in order to assess the changing patterns of the virus. We also describe the prevalence of Transmitted Drug Resistance (TDR) among this population of treatment-naïve persons.

## Methods

### Study population

From October 2003 through May 2005, blood samples were obtained from participants in two large cross-sectional surveys conducted in two adjacent rural communities (Asembo and Gem) located on the shores of Lake Victoria in western Kenya.

The aim of the surveys was to estimate the prevalence of HIV and sexually transmitted infections (STIs), and associated risk factors in rural western Kenya[10].

The majority of the inhabitants in this area are of the Luo ethnic group (98%). Together the Kenya Medical Research Institute (KEMRI) and the Centers for Disease Control and Prevention (CDC) have a longstanding presence in this region through malaria research and a comprehensive health and demographic surveillance system (HDSS) covering approximately 220,000 residents [11].

Using the health demographic surveillance system (HDSS) as a sampling platform, potential study participants were randomly selected in the first region (Asembo) through stratified sampling by sex and age group. The sampling strategies have previous been described in detail [10]. Briefly, compounds were first randomly selected from a HDSS list of housing compounds. Thereafter, one individual aged 13–34 years was selected for participation in every compound through randomly assigned numbers. From this, 1762 individuals were selected, after consenting for a blood-draw in the baseline cross-sectional survey. The second survey in the Gem Siaya District used cluster sampling (villages) to enroll 912 individuals aged 15–34 years allowing for inclusion of multiple members from the same compound. The choice of age-ranges was based on a previous survey from Kisumu by Buve et al[12]. According to preliminary findings of the Asembo cross-sectional survey, approximately 8% of adolescents initiated sexual activity before the age of 13 hence the age limit was extended to this age group. In both surveys, all participants received a review of medical history, a physical examination, voluntary testing and counseling for HIV and other STIs and pregnancy (for females). HIV status was determined from whole blood using three HIV rapid test kits as follows: Determine (Abbot Laboratories, Tokyo, Japan), Unigold (Trinity Biotech Plc, Bray, Ireland), and Capillus (Trinity Biotech Plc, Bray, Ireland) as a tiebreaker.

Treatment was provided for participants with common acute illnesses including STIs, while referrals for free tuberculosis (TB) diagnosis and treatment were made for TB suspects. Sexual partners of participants with STIs were offered free STI treatment. HIV care and support services were provided through existing infrastructure developed collaboratively between the Kenyan Ministry of Health and CDC’s Global AIDS Program[10].

### Ethical approval

Ethical approvals were obtained from the ethical review committees/institutional review boards of KEMRI/CDC, Institute of Tropical Medicine (ITM), and London School of Health and Tropical Medicine (LSHTM). Written informed consent was obtained from each participant prior to the study initiation, as previously described [10]. Minors (<18 years of age) were classified as either “mature” or “non-mature” using legal definitions. Mature minors were married or a head of a household and could consent to study participation as they could for counseling and testing in Kenya. Non-mature minors went through a two-step written consent process involving consent from the parent or guardian and a written informed assent by the minor.

### Identification of recent infections

All HIV- positive specimens were tested in parallel with the IgG BED capture enzyme immunoassay (CEIA) (Calypte Biomedical, United States) and a modified Food and Drug Administration (FDA) approved) avidity EIA test, Genetic Systems^™^ HIV-1/HIV-2 PLUS O (BioRad, Redmond, WA). These tests are calibrated to identify recent infections only at the population level. Recent infections were identified as those repeatedly concordant with both avidity and BED assays with optical density cut-off of 40% and an avidity index of 0.8, respectively. This represented a mean recency period of <239 days with the Biorad avidityand <236 days with the IgG-BED-CEIA assay[13]. All specimens with values above the described cut-off by either test were classified as long-term infection.

### Genotyping

Population-based sequencing was performed on HIV-positive specimens using an in-house assay described previously to obtain *pol* gene sequence data[14]. Sequence quality was assessed using the sequence quality assessment tool (SQUAT)[15] while contamination was checked by running controls as well as assessing intra-run sequence correlation using distance tree obtained from Phylogenetic Analysis Using Parsimony (PAUP) v4.0b[16]. Major HIV drug resistance mutations were identified using the Stanford University HIV Drug Resistance Database http://sierra2.stanford.edu/sierra/servlet/JSierra and confirmed using the International AIDS Society–USA Drug Resistance Mutations Group December 2009 update[17].

### Viral diversity, phylogenetic & recombination analysis

To determine the extent of genetic diversity, the sequences were aligned using multiple sequence alignment programs in Molecular Evolutionary Genetics Analysis (MEGA) software v5[18] to obtain 877 nucleotide bases devoid of gaps. Phylogenetic relationship was then determined using Neighbor-Joining method in MEGA v7 and confirmed with REGA HIV-1 v3.0[19]. Simplot v3.5.1[20] was then used for the analysis of recombinant sub-types using a 400 base pair (bp) window with a 20 bp increment. Differences in viral diversity between recent and long-term viruses were assessed by reviewing the distribution of subtypes and recombinants by group—based on recency of infection.

### Identification of transmission networks

Sequence relatedness was estimated using neighbor joining trees and maximum likelihood methods integrated in Mega v 7.0. Potential transmission clusters were ascertained using robust statistical criterion of the maximum likelihood topologies assessed by high bootstrap values (≥85%)[21] with 1000 resamplings and short genetic distances (Tamura Nei 93 distances) of ≤0.015[1,21,22]. The trees were rooted using subtype K reference sequence (Los Alamos Database accession number AJ249239_CM_K).

Characteristics of the transmission networks were analyzed by comparing demographics and behavioral information of survey participants in the network versus those out of the network.

### Statistical analysis

Participant baseline characteristics were described using percentages for categorical data and median and interquartile ranges (IQR) for continuous data. Logistic regression was used to identify independent factors associated with recent infection. Variables that were significant at an alpha level of 0.25 were then entered into the multivariable analysis.

Differences in subtype distribution between recent and long-term infections were assessed by Pearson chi-square test.

## Results

Among the 2674 persons aged 13–34 years who enrolled in the surveys, 48% were male and 52% were female. HIV seroprevalence was 14.6% (95%CI 13.3–16.0), with no significant difference between the two communities of GEM (15.1%) and Asembo (14.8%). This analysis included 398 HIV seropositive subjects, among whom 261 (65.6%) were females and 137 (34.4%) males, representing a 2:1 ratio.

### Recent infections

Out of 398 HIV specimens from HIV seropositive participants, 320 had adequate sample volume to be tested for recent and long-term HIV infection. Of these specimens, 40 were concordantly identified as recent infection by both the IgG BED-CEIA and the modified FDA-approved avidity EIA assays. The majority of the recent infections identified were among females (70%), most of whom (12 of 28; 47%) were in the 15–19 age group (Table 1). Factors significantly associated with recent infections, based on the bivariate analysis, were age ≤19 years versus older age [odds ratio (OR) 7.67 (95% confidence interval (CI) 3.66–16.05)], being single/never married [OR 4.38 (95% CI 2.07–9.26)], having sex with a person other than the primary partner in last six months [OR 2.24 (95% CI 1.07–4.68)] and history of STI in the past six months [OR 41.67 (95% CI 6.62–261.98)] (Table 2). In the multivariable analysis only younger age (participants ≤19 years) [adjusted OR (aOR) 4.85 (95%CI 1.99–11.83)] and a history of STIs in the past six months [aOR 51.7 (95%CI 3.6–733.10] remained significantly associated with testing recent (Table 2).

**Table 1:** Characterization of recent and long-term HIV infections by age and gender, Western Kenya, October 2003 –May 2005.

Total n = 320	Recent infections n = 40 (12.5%)		Long-term infections n = 280 (87.5%)	
Age group	Females n = 28 (70%)	Males n = 12 (30%)	Females n = 178 (63.6%)	Male n = 102 (36.4%)
13–14	1 (3.6)	1 (8.3)	1 (0.6)	_
15–19	12 (42.9)	4 (33.3)	32 (18.0)	3 (2.9)
20–24	5 (17.9)	6 (50.0)	28 (15.7)	6 (5.9)
25–29	6 (21.4)	1 (8.3)	61 (34.3)	44 (43.1)
30–34	4 (14.3)	-	56 (31.5)	49 (48.0)

The frequency of both recent and long-term infections was higher among female as compared to males. The highest proportion of recent infections was observed among females aged 15–19 (12/40 30%).

**Table 2:** Unadjusted and Adjusted Odds ratios and 95% CI for factors associated with recent infections among HIV-1 positive participants in Western Kenya, October 2003 –May 2005.

	*Long-term infections*	*Recent infections*	*Bivariate*		*Multivariate*	
N	280	40	OR (95% CI)	P-value	OR (95% CI)	P-value
Gender						
Male	102	12	1.0	0.490	_	_
Female	178	28	1.29 (062–2.65)			
Marital status						
Married	181	18	1.0			
Divorced/separated	11	1	0.91(0.11–7.50)	0.822		
Single	39	17	***4*.*38(2*.*07–9*.*26****)*	***<0*.*0001***	1.69(0.65–4.41)	0.281
Widowed	49	4	0.82(0.27–2.54)	0.732		
Age						
>19	253	22	1.0		1.0	
≤19	17	18	***7*.*67(3*.*66–16*.*05)***	***<0*.*0001***	***4*.*85(1*.*99–11*.*83)***	***0*.*001***
Sex in past 6 months with person other than partner						
No	223	24	1.0		1.0	
Yes	54	13	***2*.*24(1*.*07–4*.*68)***	***0*.*032***	1.68(0.70–4.04)	0.243
Condom use	26	2	1.0			
No	199	22	1.44(0.32–6.47)	0.636		
Yes						
History of STI						
No	202	32	1.0		1.0	
Yes	78	8	0.65(0.29–1.47)	0.241	0.69(0.28–1.69)	0.412
History of STI in past 6 months						
No	75	3	1.0			
Yes	3	5	***41*.*67(6*.*62–261*.*98)***	***<0*.*0001***	***51*.*68(3*.*64–733*.*10)***	***0*.*004***
More than one spouse						
No	148	13	1.0			
Yes	34	5	1.67(0.56–5.01)	0.285		
No. of spouses						
0	13	3	1.0			
1	5					
2	11	2	0.79(0.11–5.56)	0.812		
3	5					

### Phylogenetic and recombinant analysis

Of the 398 samples, 312 were genotyped, of which 258 (82.7%) yielded a sequence and were successfully analyzed. Of these, the subtype results were as follows: 171 (66.3%) A, 36 (14.0%) D, 18 (7.0%) C, 1 (0.4%) G, and 32 (12.4%) recombinants. The majority of inter-subtype recombinants were A/D (59%), followed by CRF10 (25%), CD (9%) and A/C (6%). Sequences analyzed in this study have been submitted to GenBank (accession numbers KU248496-KU248753)

### Subtype distribution in those testing recent versus long-term infection

**Fig 1:**
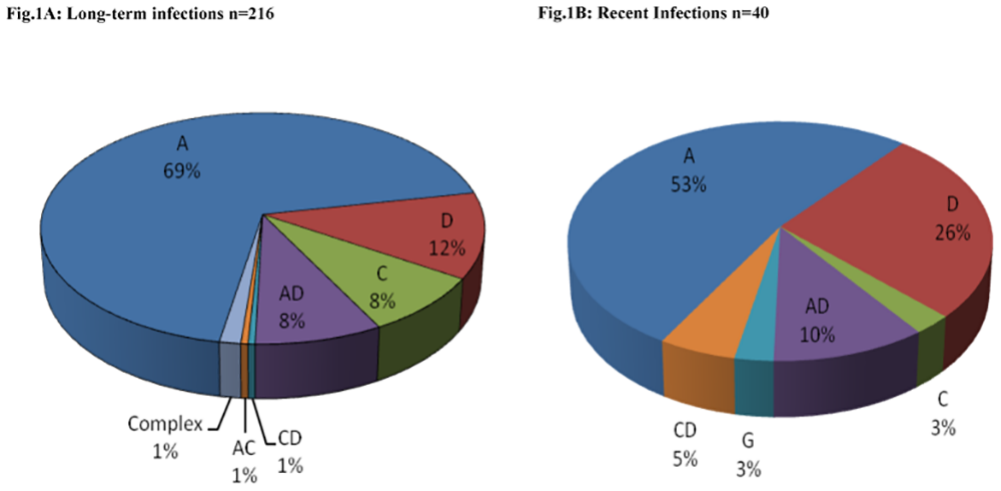
Distribution of HIV-1 subtypes among participants, by recency of infection, Western Kenya, October 2003-May 2005. **(A)** Long term (>239 days) (B) recent infections (≤239 days) (Fig 1B). A, C, D, G represent pure subtypes while AD, AC, CD are unique recombinant forms, CRF; Complex recombinants forms.

Comparison of subtype distribution among persons with recent and long-term infection revealed that subtype A dominates in both groups but with a significant higher proportion in long-term 68% vs recent infections 53% (*p = 0*.*04)*. Subtype D was, by contrast, significantly more common in the recent versus the long-term group (26% vs. 12%, p = 0.012), while an almost equal distribution of subtype C and recombinant strains observed among the two groups. The unique A/C recombinant (1%) was identified within the long-term infection group while subtype G existed only within the recently infected group (Fig 1).

### HIV drug resistance mutations

Of the 258 successfully genotyped samples, primary drug resistance mutation was detected in three (1.16%) samples with M41L being the only detected mutation.

### Assessment of cluster characteristics

**Fig 2:**
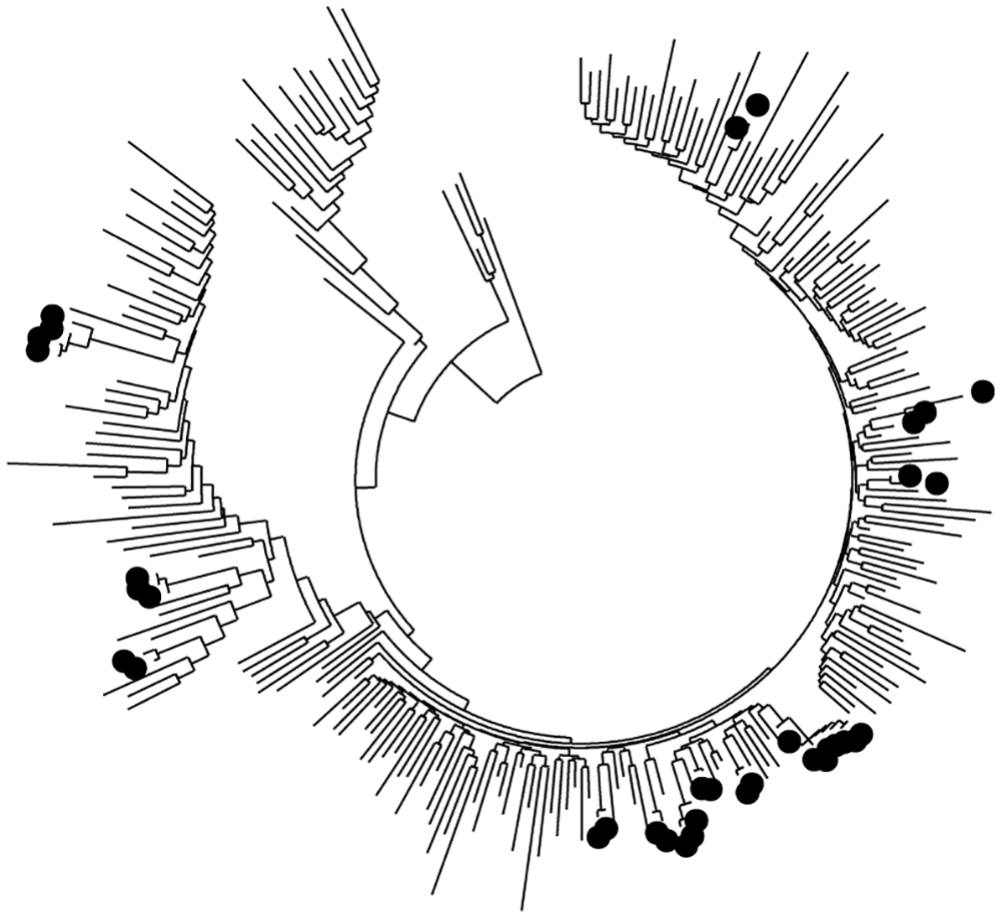
HIV-1 transmission clusters among heterosexuals in the Gem and Asembo region of Western Kenya. Phylogenetic tree showing HIV-1 transmission clusters in the Gem and Asembo region. The highlighted tree nodes represent members in transmission clusters with bootstrap values of 85% and mean genetic distance of 0.015-nucleotide substitution per site. Sequences with drug resistance mutation are highlighted with grey. The clusters correlate with the demographic information shown in table 4 in a clockwise phase (Cluster 1 through 12).

Of the 258 sequences, 29 (11.2%) fell into 12 monophyletic clusters, each comprising 2–4 members (Fig 2).

Nine of the clusters were of subtype A, and three of subtype D. The majority of the clusters were confined within one geographical location, with only two clusters spanning the two regions. In univariate analysis individuals in the network were more likely to be recently infected [OR 3.88 (95% CI 1.59–9.51)] of age ≤19 years [OR 2.73 (95% CI 1.14–6.54)] being single/never married [OR 2.71 (95% CI 1.09–6.72)] and having sex with a person other than the primary partner in last six months [OR 2.56 (95% CI 1.12–5.85)]. In multivariate analysis being recently infected [OR 2.93 (95% CI 1.04–8.30)] and being from Gem region [OR 4.31 (95% CI 1.65–11.25)] remained independently associated with clustering (Table 3).

**Table 3:** Unadjusted and Adjusted Odds ratios and 95% CI for factors associated with transmission clusters among HIV-1 positive participants in Western Kenya.

	*All participants*	*In Networks*	*Bivariate*		*Multivariate*	
N	258	29	OR (95% CI)	P-value	OR (95% CI)	P-value
Gender						
Male	79	12	1.0	0.375		
Female	151	16	0.70(0.31–1.55)			
Marital status						
Married	149	15	1.0			
Divorced/separated	40	2	0.50(0.11–2.26)	0.366		
Single	33	9	***2*.*71(1*.*09–6*.*72)***	***0*.*032***	1.82(0.55–6.02)	0.327
Widowed	8	2	2.48(0.48–12.78)	0.276		
Age						
>19	196	34				
≤19	19	9	***2*.*73(1*.*14–6*.*54)***	***0*.*024***	1.28(0.41–3.98)	0.669
Infection recency						
Long term	205	25	***1*.*0***			
Recent	19	9	***3*.*88 (1*.*59–9*.*51)***	***0*.*003***	***2*.*93(1*.*04–8*.*30)***	***0*.*043***
Region						
Asembo	144	7	***1*.*0***			
Gem	86	22	***5*.*02(2*.*05–12*.*31)***	***<0*.*001***	***4*.*31(1*.*65–11*.*25***	***0*.*002***
Sex in past 6 months with person other than partners						
No	178	17	1.0			
Yes	45	11	***2*.*56(1*.*12–5*.*85)***	**0.026**	1.53(0.53–4.37)	0.428
History of STI						
No	167	17	1.0			
Yes	59	11	1.83(0.81–4.14)	0.145	1.79(0.70–4.58)	0.227
More than one spouse						
No	117	13	1.0			
Yes	29	2	0.62(0.13–2.90)	0.545		

33% of the clusters were from female participants and a similar proportion for persons >19 years. All but one cluster involving participants’ ≤19 years had members >19 years (Table 4).

**Table 4:** Demographic characteristics of HIV-infected participants, by genetic cluster of HIV-1 strains, Western Kenya, October 2003 –May 2005.

Transmission cluster	Participant ID	Subtype	Gender	Age	Marital Status	Location	Infection status*
1.	001^A^	A	Female	25	Married	Gem	LT
	002^A^		Male	29	Married	Gem	LT
2.	003	A	Male	28	Married	Gem	LT
	004		Male	20	Married	Gem	LT
3.	005	A	Female	18	Married	Gem	LT
	006		Female	21	Married	Gem	LT
4.	007	A	Male	20	Married	Asembo	LT
	008		Female	19	Not married	Gem	LT
5.	009^B^	A	Female	26	Married	Gem	LT
	010		Female	18	Not married	Gem	Recent
	011^B^		Male	34	Married	Gem	LT
6.	022	A	Female	18	Married	Gem	Recent
	023		Female	17	Not married	Gem	Recent
7.	014	A	Male	28	Married	Asembo	LT
	015		Male	34	Not married	Asembo	LT
8.	016		Female	19	Married	Asembo	Recent
	017	A	Female	26	Not married	Asembo	LT
	018		Male	18	Not married	Gem	LT
9.	019	A	Male	25	Not married	Gem	LT
	020		Female	25	Married	Gem	LT
	021		Male	19	Not married	Gem	LT
10.	022	D	Female	17	Not married	Asembo	Recent
	023		Female	20	Married	Asembo	LT
11.	024	D	Female	25	Not married	Gem	LT
	025		Female	25	Not married	Gem	LT
12.	026	D	Female	31	Married	Gem	LT
	027^C^		Female	23	Married	Gem	LT
	028^C^		Male	24	Married	Gem	LT
	029		Male	15	Not Married	Gem	LT

* LT-long term

^A,B,C^ Participants from same house, possibly couples

Two of the participants with primary drug resistance mutations were in the clusters as a dyad (Fig 2). A further assessment based on the sampling details revealed that these participants were from the same house (cluster table 4). Similarly cluster four and two had participants from the same house.

## Discussion

In this study, we have described the molecular characteristics of the HIV-1 epidemic in rural western Kenya, with specific focus on recent infections, HIV-1 genetic diversity, transmission networks, and transmitted HIV drug resistance. In an earlier study we showed that HIV infection in this population was associated with younger age, higher number of sex partners, widowhood and HSV-2 seropositivity[10]. In this study we further assessed the correlates for recent infections and transmission clusters. Identifying characteristics associated with recent infections provides important information on the dynamics of the epidemic, vital in guiding public health intervention programs. Using laboratory-based methods, 12.5% of the participants assessed had recent infections, the majority of whom were females. The high proportion of females with recent infections is consistent with gender distribution for HIV prevalence observed in this population as well as with that observed from other studies in Kenya [10,23,24]. Equally the young age of recently infected females suggests the vulnerability of late adolescent females for acquiring HIV infection. This might be due to the early sexual debut of females in this population as well as their involvement with older men who are more likely to be infected. Age mixing in sexual relations has been reported in sub-Saharan Africa, with a higher risk of HIV infections among females having sexual partners who are ten or more years older[24–26]. Comparatively, both long-term and recent HIV infection was lower in young males than in young females, suggesting a lower risk of infection as compared to their female counterparts.

In general, younger age was identified as independently associated with recent infection. This suggests that despite the low prevalence of HIV among the young males, they are still at risk of getting new infections just like their female counterparts.

Recent history of STI was another independent factor associated with recent infections. While certain STIs have been identified as significant risk factors for acquiring or transmitting HIV infection, evidence for and utilization of prevention efforts based on early identification and treatment of STIs has generally been weak in sub-Saharan Africa[27–29]. Nonetheless, this is a reminder that symptomatic STIs and HIV travel hand-in hand, and highlights the need for expanding STI testing and treatment services as part of HIV epidemic response.

As with previous studies from Kenya, our study documents the predominance of HIV-1 subtype A, followed by subtype D, and a lower prevalence of subtypes C, AD recombinants and G variants[14,30,31]. The presence of recombinant viruses, with predominance of A/D forms, has previously been reported in western Kenya[14]. In this study, 12.4% of the circulating strains were recombinants, with a predominance of AD variants. A single subtype G was identified in a 16 year old recently infected female. Subtype G strains are known to circulate in Kenya but in very low numbers[14,30,31].

Subtype distribution between long-term and recently infected persons showed variation, with subtype A viruses predominating in both groups but being significantly higher among the long-term infections, compared to the recent infections. Subtype D was higher among the recent infections as compared to long-term infections. A significant increase in subtype D strains among the recent infections, with a concomitant decrease in A variant, may be an indication of an epidemic shift. This expansion of subtype D epidemic is likely to have negative implications due to the observed high virulence and faster disease progression of the D strain as compared to subtype A variants. The virulent nature of subtype D is believed to be due to its ability to use both the CXCR4 and CCR5 receptors simultaneously throughout the infection and as a result being associated with faster disease progression[32,33].

Despite the high number of subtype D strains among the recent infections, the number of A/D recombinant forms was equally distributed among recent and long-term infections, which may be suggestive of a consistent continuous mixing of the two strains in the study population.

The relatively low level of primary TDR (only 3 specimens) was expected in a population of ARV-naïve individuals from a rural area of Kenya in a time when widespread antiretroviral therapy was just beginning. The M41L (a thymidine analogue mutation) mutation observed in the RT gene is known to appear as a natural variant, causing low-level resistance to zidovudine (AZT) and stavudine (d4T) and potential low-level resistance to didanosine (DDI), abacavir (ABC) and tenofovir (TDF). A combination of M41L and the T215Y TAMs however results with high-level resistance to AZT and d4T and intermediate level resistance to DDI, ABC and TDF^31^.

In general we observed a low TDR threshold (<5%), which suggests that the recommended first-line regimen was optimal for use in this population at the time of the study (2003–2005). However, this scenario is likely to have changed with the mass rollout of free ARV drugs, and there is need for recent evaluation and continuous monitoring of drug resistance in this region.

Twelve transmission clusters comprising twenty-nine individuals were identified in this study, eight of which were dyads. The observation of clusters confined within a community may suggest that the identified transmission networks have true epidemiological linkages. The majority of the clusters were predominantly among females, which may suggest common risks for HIV among females in these communities.

Furthermore, nearly a third of the individuals in the clusters were young persons of <19 years although the majority of the clusters had a higher median age (>19 years). This clustering, together with existing inter-age mixing of sex partners, suggests the vulnerability of young persons for HIV infection and highlights the need for prevention strategies focused on the younger population. The occurrence of small component networks and dyads which mainly involve older married persons is suggestive of concurrent relationships involving extra-marital affairs. Involvement of married persons in such networks corroborates the Kenya AIDS indicator survey report, which showed a high HIV prevalence among married persons, suggesting the need to consider tailoring prevention efforts on this group as a nationwide strategy against HIV infection[24]. Consistent with other molecular epidemiology studies, was the observed association of recently infected cases with clustering[22,34]. This and other findings provide growing evidence suggesting the potential impact of early treatment in the prevention of HIV transmission [22,34–36].

The observed higher number of clusters from Gem may partially be explained by the difference in sampling between the two studies. In Asembo compounds were randomly selected from a list of housing compounds and only one individual per compound was asked to participate. In Gem however compounds were randomly selected from randomly selected villages and all members in the compound were invited to participate. This may have resulted with more members from close villages being enrolled in the study; hence more persons in the clusters especially if the sexual networks are confined within close proximities. Moreover from the data three pairs in the networks were from the same house, and all were from Gem region.

Study limitations exist. Our use of serological assays to identify age of infection without confirmation by use of viral-load measurements may have potentially included false recent infections.

There is also likelihood for incomplete network bias as the study population included only persons of age 13–34 years and may have missed potential key components of the network if persons beyond 34 years are part of these sexual networks.

It is, however, unclear how this exclusion would have affected the validity of the observed networks. In addition, the lack of qualitative behavioral assessment did not allow for a more in-depth assessment of the role of high-risk groups including IDU in the HIV epidemic in this region. Although the prevalence of IDU in Kenya is higher among the inhabitants of the coastal regions, a recent study showed the existence of HIV-infected IDU’s in western Kenya[37]. In this study no information was collected concerning IDU’s, and this limits an in-depth description of all the possible mechanism of HIV transmission through the networks.

Moreover, the study findings are mainly a reflection of the early ART period (2003–2005). It’s likely that the levels of TDR have changed, given the increased use of ART in this region. In addition, various prevention strategies have been initiated and could have affected the nature and structure of the observed social networks. It’s important to note that HIV incidence and prevalence have remained high in this region[24] and it’s possible that such networks may still be in existence and playing a significant role in the spread of HIV. A multi-disciplinary approach to analysis is needed to fully determine and address the spread of HIV among sexual networks.

Despite these limitations, this study gives an insight in the epidemiology of HIV infection in two rural communities in western Kenya highlighting a higher risk of HIV transmission in young people whose needs remain neglected and for whom targeted services could substantially reduce HIV transmission. Our findings also indicate the utility of molecular data in describing the epidemiology of the HIV epidemic, with specific focus on recent infection and transmission dynamics. More recent data is required to further elucidate the current status of the networks and identify appropriate effective and targeted HIV prevention interventions.

## Disclaimer

The findings and conclusions in this article are those of the authors and do not necessarily represent the views of the U.S. Centers for Disease Control and Prevention. Use of trade names is for identification purposes only and does not constitute endorsement by the U.S. Centers for Disease Control and Prevention or the Department of Health and Human Services.
